# Protocol for enhancing Cas9 efficiency and fidelity through structure-guided phosphate-locking loop engineering

**DOI:** 10.1016/j.xpro.2026.104452

**Published:** 2026-03-25

**Authors:** Meiying Yang, Guanqiao Chen, Ji Xiao, Xin Zhang, Zheng Hu, Bo Zhou

**Affiliations:** 1 Department of Gynaecology, Guilin People’ S Hospital, 12 Wenming Road, Xiangshan District, Guilin, Guangxi 541002, China; 2 Taikang Center for Life and Medical Sciences, Wuhan University, No.299 Bayi Road, Wuchang District, Wuhan, Hubei 430072, China; 3 Department of Gynecologic Oncology, Women and Children’s Hospital Affiliated to Zhongnan Hospital of Wuhan University, Wuhan, Hubei 430071, China; 4 Hubei Key Laboratory of Tumor Biological Behavior, Hubei Provincial Clinical Research Center for Cancer, Zhongnan Hospital of Wuhan University, Wuhan, Hubei 430071, China

**Keywords:** Genomics, Sequencing, Molecular Biology, CRISPR, Cryo-EM

## Abstract

The phosphate-locking loop (PLL), stabilizing Cas9-DNA interactions, is a key target for optimizing efficiency and specificity. Here, we present a protocol for enhancing Cas9 efficiency and fidelity through structure-guided PLL engineering. We describe steps for identifying PLL engineering targets through sequence alignment and structural analysis, constructing variants via inverse PCR, evaluating efficiency using amplicon sequencing, and assessing specificity through Genome-wide Unbiased Identification of DSBs Evaluated by sequencing (GUIDE-seq (GUIDE-seq). This protocol provides a generalizable framework for Cas9 engineering across orthologs.

For complete details on the use and execution of this protocol, please refer to Yang et al.^1^

## Highlights

• Steps for identifying PLL engineering targets via alignment and structural analysis
• Instructions for constructing Cas9 variants through structure-guided mutagenesis
• Guidance on assessing efficiency by amplicon sequencing and specificity by GUIDE-seq
• Steps for engineering Cas9 orthologs using PLL design principles

## Abstract (editor highlights)

Publisher’s note: Undertaking any experimental protocol requires adherence to local institutional guidelines for laboratory safety and ethics.

## Graphical abstract

**Figure:**
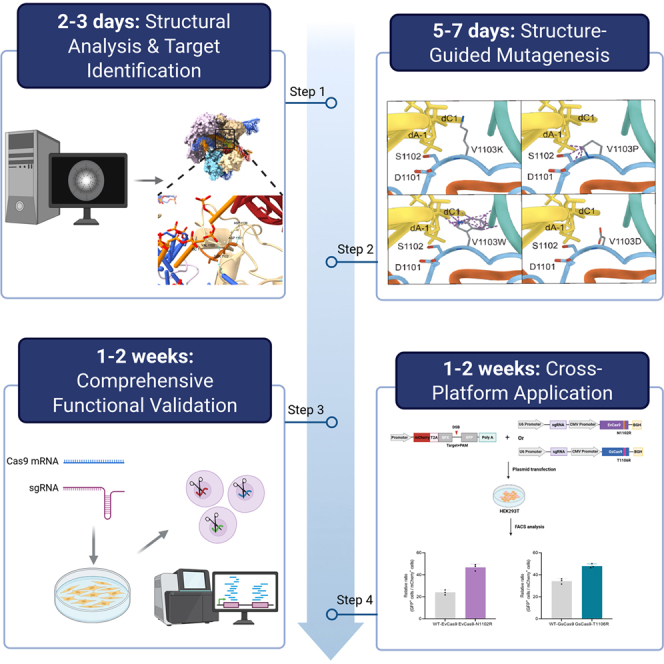

## Teaser

The phosphate-locking loop (PLL), stabilizing Cas9-DNA interactions, is a key target for optimizing efficiency and specificity. Here, we present a protocol for enhancing Cas9 efficiency and fidelity through structure-guided PLL engineering. We describe steps for identifying PLL engineering targets through sequence alignment and structural analysis, constructing variants via inverse PCR, evaluating efficiency using amplicon sequencing, and assessing specificity through GUIDE-seq. This protocol provides a generalizable framework for Cas9 engineering across orthologs.

## Before you begin

In the following protocol, we describe a structure-guided approach to engineer the phosphate-locking loop (PLL) domain in CRISPR-Cas9 nucleases for enhanced editing fidelity. Using FrCas9 as a model system, cryo-EM structural analysis identified an electron density cavity at residue V1103 within the D1101-S1102-V1103 PLL motif. Detailed structural characterization revealed that D1101 and S1102 form multiple hydrogen bonds and salt bridges with the +1 phosphate of the target DNA strand, while.

V1103 contributes minimal direct contact—presenting an engineering-amenable cavity suitable for introducing beneficial interactions.

We performed comprehensive mutagenesis at FrCas9 V1103, testing all 19 amino acid substitutions to systematically map structure-activity relationships. This revealed that extended, positively charged residues (K, R) optimally enhance both phosphate backbone interactions and editing fidelity, with V1103K achieving 30.15% improvement across nine target sites. Structural analysis of successful and unsuccessful variants established key design principles: optimal substitutions introduce favorable electrostatic interactions while maintaining appropriate spatial geometry within the cavity, whereas aromatic residues cause steric clashes and negatively charged residues disrupt DNA binding through electrostatic repulsion.

These structure-activity insights, derived from FrCas9 characterization, inform rational engineering of analogous PLL positions in other Cas9 systems. The protocol integrates structural analysis of protein-DNA interfaces—including hydrogen bonding patterns, salt bridge formation, cavity geometry, and electrostatic complementarity—with functional validation through genome editing assays and high-throughput sequencing. This approach has been successfully applied to EvCas9 and GsCas9, demonstrating broad applicability across Type II-A Cas9 orthologs with conserved PLL architecture.^2^^,^^3^^,^^4^^,^^5^^,^^6^

### Innovation

This protocol presents the first systematic engineering of the phosphate-locking loop (PLL) motif in Cas9 nucleases—a critical structural element stabilizing Cas9-DNA interactions that was previously underexplored as an optimization target. Unlike existing approaches that focus on modifying REC domains or DNA binding regions to reduce off-target activity (often at the cost of on-target efficiency), PLL engineering simultaneously enhances both specificity and editing efficiency by strengthening DNA backbone interactions.

The key innovation lies in identifying the PLL as an engineering-amenable target through structural cavity analysis. In FrCas9, the D1101-S1102-V1103 motif contacts the +1 phosphate, with V1103 residing in a unique electron density cavity that allows substitution with larger residues. This structural feature enables introduction of positively charged side chains (K, R) that enhance electrostatic interactions without disrupting the essential contacts formed by flanking residues—a design space unavailable in tightly packed protein-DNA interfaces.

The protocol achieves 30.15% fidelity improvement in FrCas9 while maintaining robust editing efficiency, addressing a key limitation of existing high-fidelity variants that often sacrifice activity for specificity. The strategy proves broadly applicable across Type II-A Cas9 orthologs (validated in EvCas9 and GsCas9), providing a generalizable framework for improving CRISPR nucleases without extensive protein engineering infrastructure. This represents a novel engineering paradigm targeting phosphate backbone stabilization rather than DNA sequence recognition.

## Key resources table

**Table:** 

REAGENT or RESOURCE	SOURCE	IDENTIFIER
**Bacterial and virus strains**		

Trans5a Chemically Competent Cell	Trans	Cat#CD201-01
Competent DH5α Cell	Tsingke Biotech	Cat#DLC114-100

**Experimental models: Cell lines**		

HEK293T cells	ATCC	CRL-3216

**Chemicals, peptides, and recombinant proteins**		

DMEM, high glucose	Gibco	Cat#11965092
Fetal bovine serum (FBS), heat-inactivated	Gibco	Cat#10270106
Penicillin-Streptomycin (100×)	Gibco	Cat#15140122
Trypsin-EDTA (0.25%), phenol red	Gibco	Cat#25200056
Phosphate-buffered saline (PBS), pH 7.4	Gibco	Cat#10010023
Opti-MEM Reduced Serum Medium	Gibco	Cat#31985062
SOC outgrowth medium	New England Biolabs	Cat#B9020S
LB Broth (Lennox)	Sigma-Aldrich	Cat#L3022
LB Agar (Lennox)	Sigma-Aldrich	Cat#L2897
Ampicillin sodium salt	Sigma-Aldrich	Cat#A9518
Kanamycin sulfate	Sigma-Aldrich	Cat#K1377

**Critical commercial assays**		

EasyPure® Genomic 621 DNA Kit	Transgen	Cat#TEE101-01
2xPhanta Max Master Mix	Vazyme	Cat#P515-01
2X MultiF Seamless Assembly Mix	ABclonal	Cat#RK21020
QIAGEN Plasmid Midi Kit	Qiagen	Cat#12143
DNeasy Blood & Cell Culture DNA Kit	Qiagen	Cat#69506
QIAquick Gel Extraction Kit	Qiagen	Cat#28706
QIAquick PCR Purification Kit	Qiagen	Cat#28106
GUIDE-seq Kit	Horizon Discovery	Cat#GE-100020
NEBNext Ultra II DNA Library Prep Kit for Illumina	New England Biolabs	Cat#E7645S
NEBNext Multiplex Oligos for Illumina (Index Primers Set 1)	New England Biolabs	Cat#E7335S
Qubit dsDNA HS Assay Kit	Thermo Fisher Scientific	Cat#Q32851
Agilent High Sensitivity DNA Kit	Agilent Technologies	Cat#5067-4626
AMPure XP beads	Beckman Coulter	Cat#A63881
Hieff Trans® Liposomal Transfection Reagent	Yeasen	Cat#40802ES08
TIANcombi DNA Lyse&Det PCR Kit	Tiangen	Cat#KG203-02
2× Phanta Max Master Mix (Dye Plus)	Vazyme	Cat# P525-01

**Oligonucleotides**		

V1103 saturation mutagenesis primers (see Table S1 for sequences)	Integrated DNA Technologies (IDT)	Custom synthesis
GUIDE-seq dsODN tag (34 bp)	Integrated DNA Technologies (IDT)	Custom synthesis
Amplicon sequencing primers (see Table S2 for sequences)	Integrated DNA Technologies (IDT)	Custom synthesis
Sanger sequencing primers for clone verification	Integrated DNA Technologies (IDT)	Custom synthesis

**Recombinant DNA**		

pFrCas9 wild-type expression plasmid	This study	Available upon request
pFrCas9-V1103K variant plasmid	This study	Available upon request
pFrCas9-V1103R variant plasmid	This study	Available upon request
pX330-U6-Chimeric_BB-CBh-hSpCas9 (sgRNA expression vector)	Addgene	Plasmid #42230
pCMV-EGFP (transfection efficiency control)	Clontech	Cat#6085-1

**Software and algorithms**		

Amplicon sequencing analysis	CRISPResso2	http://crispresso.pinellolab.partners.org/
GUIDE-seq analysis pipeline	guideseq	https://github.com/aryeelab/guideseq
sgRNA design	Benchling	https://www.benchling.com
Primer design	Primer3	https://primer3.ut.ee/
Oligonucleotide analysis	IDT OligoAnalyzer	https://www.idtdna.com/calc/analyzer
Protein cavity analysis	CASTp 3.0	http://sts.bioe.uic.edu/castp/
Multiple sequence alignment	Clustal Omega	https://www.ebi.ac.uk/Tools/msa/clustalo/
Structure prediction	AlphaFold3	https://alphafold.ebi.ac.uk/
Protein engineering suite	FoldX 5.0	http://foldxsuite.crg.eu/
Electrostatic calculations	APBS	https://www.poissonboltzmann.org/
Structure validation	MolProbity	http://molprobity.biochem.duke.edu/
Statistical analysis and graphing	GraphPad Prism 9	https://www.graphpad.com
Statistical computing	R version 4.2.0	https://www.r-project.org/
Sequence similarity search	BLAST	https://blast.ncbi.nlm.nih.gov/
Sequencing data quality control	Fastp 0.20.0	http://www.imeta.science
Paired-end read merging	FLASH	https://academic.oup.com/bioinformatics
Molecular structure visualization	ChimeraX 1.4	https://rbvi.ucsf.edu/chimera/download.html

**Other**		

SF Cell Line 4D-Nucleofector X Kit	Lonza Scientific	Cat# V4XC-2024
Illumina NextSeq 550 System	Illumina	Cat#SY-415-1002
NextSeq 500/550 High Output Kit v2.5 (150 Cycles)	Illumina	Cat#20024907
Qubit 4 Fluorometer	Thermo Fisher Scientific	Cat#Q33226
Agilent 2100 Bioanalyzer System	Agilent Technologies	Cat#G2939BA
NanoDrop One Spectrophotometer	Thermo Fisher Scientific	Cat#ND-ONE-W
T100 Thermal Cycler	Bio-Rad	Cat#1861096
DynaMag-2 Magnetic Rack	Thermo Fisher Scientific	Cat#12321D
Centrifuge 5424 R (refrigerated microcentrifuge)	Eppendorf	Cat#5404000510
Forma Series II 3110 Water-Jacketed CO_2_ Incubator	Thermo Fisher Scientific	Cat#3110
Forma Class II, Type A2 Biosafety Cabinet	Thermo Fisher Scientific	Cat#1300 Series
Countess II Automated Cell Counter	Thermo Fisher Scientific	Cat#AMQAX1000
PowerPac Basic Power Supply	Bio-Rad	Cat#1645050
Mini-Sub Cell GT Horizontal Electrophoresis System	Bio-Rad	Cat#1704467
ChemiDoc MP Imaging System	Bio-Rad	Cat#12003154
24-well plate	BIOFIL	Cat#TCP010024

## Step-by-step method details

### PLL motif identification and structural analysis

**Timing: 2–3 days**

This section identifies engineering targets within the Cas9 phosphate-locking loop (PLL) domain through sequence alignment and structural analysis. This approach is applicable to any Cas9 ortholog with available structural data. FrCas9 is used as a worked example throughout.

1. PLL Motif Identification via Sequence Alignment.
  a. Obtain Cas9 protein sequences from UniProt (https://www.uniprot.org/) or NCBI (https://www.ncbi.nlm.nih.gov/):
    i. The target Cas9 ortholog of interest.
    ii. Reference sequences with characterized PLL elements: FrCas9 (PLL: D1101-S1102-V1103) and SpCas9 (PLL: K1107-E1108-S1109).
  b. Perform multiple sequence alignment using Clustal Omega (https://www.ebi.ac.uk/Tools/msa/clustalo/) or MUSCLE:
    i. Upload sequences in FASTA format.
    ii. Use default parameters for global alignment.
    iii. Export alignment in Clustal format for visualization.
  c. Identify the PLL motif in the target Cas9:
    i. Locate the region aligning to FrCas9 residues 1095-1110.
    ii. Identify the three-residue motif corresponding to FrCas9 D1101-S1102-V1103.
    iii. Record the residue numbers and native amino acids at these positions in the target Cas9.
  d. Evaluate sequence conservation in the PLL region:
    i. High conservation (>60% identity): Cross-platform engineering strategy is highly reliable.
    ii. Moderate conservation (40**–**60%): Additional structural validation is recommended.
    iii. Low conservation (<40%): Extensive structural analysis is recommended before engineering.
2. Structural Analysis of PLL Motif.
  a. Obtain the crystal or cryo-EM structure of the target Cas9:
    i. Download from PDB database (https://www.rcsb.org) if available (preferred).
    ii. If unavailable, generate AlphaFold3 prediction for the Cas9-sgRNA-DNA ternary complex.
    iii. For AlphaFold3 predictions, validate quality: PAE <5 Å between protein and DNA regions; DNA backbone maintains proper B-form geometry.
      ***Note:*** For FrCas9, the crystal structure is available as PDB ID 9JWK.
  b. Load the structure into ChimeraX v.1.4 visualization software. And locate the PLL element identified in step 1:
    i. Navigate to the residues identified through sequence alignment.
    ii. Confirm proximity to the +1 phosphate of the target DNA strand.
      ***Note:*** For FrCas9, residues D1101, S1102, and V1103 are interacted with the +1 phosphate of the target DNA strand.
  c. Upload the PDB file to PISA server (https://www.ebi.ac.uk/pdbe/pisa/) and select “Interfaces” analysis for protein-DNA interactions.
  d. Review PISA output to identify specific interactions for each PLL residue:
    i. Hydrogen bonds: Direct or water-mediated H-bonds with DNA phosphate groups.
    ii. Salt bridges: Electrostatic interactions between charged residues and phosphate backbone.
    iii. Hydrophobic contacts: Van der Waals interactions contributing to binding stability.
    iv. Interface area: Buried surface area contributed by each residue.
  e. Document quantitative metrics from PISA for the identified PLL residues:
    i. Number of hydrogen bonds per residue.
    ii. Distance and geometry of salt bridges.
    iii. Interface area contribution (Ų).
    iv. Identify which residues are primary DNA contacts (>2 hydrogen bonds, significant interface area contribution) versus potential engineering targets (<1 hydrogen bond, minimal direct contact but spatially positioned near phosphate backbone).
      ***Note:*** For FrCas9, comparative analysis shows S1102 forms direct hydrogen bonds (strongest); D1101 contributes larger buried surface area; V1103 shows fewer polar interactions and smaller interface area relative to D1101 and S1102, identifying V1103 as the engineering target (Figures 1A and 1B).
      **Figure 1:**
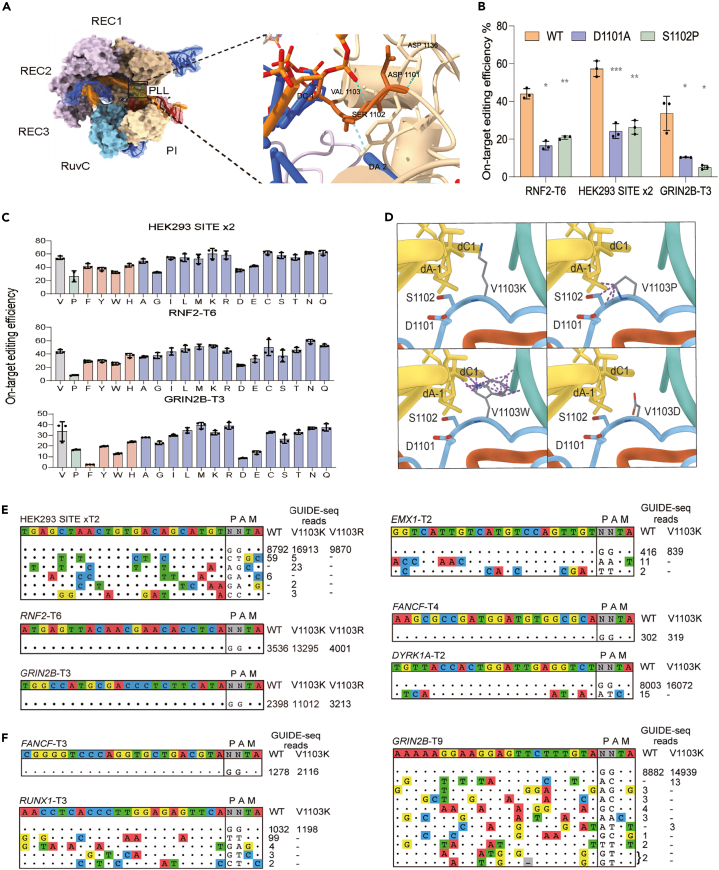
Functional Characterization of FrCas9 V1103 Saturation Mutagenesis (A) Crystal structure of FrCas9 PLL domain showing D1101-S1102-V1103 motif with the structural cavity at V1103 highlighted. (B) On-target editing efficiency for two single amino acid mutation variants of FrCas9 (D1101A and S1102P) with three sgRNAs. Data represent mean ± SEM from n=3 biological replicates. ∗*p* < 0.05, ∗∗*p* < 0.01, ∗∗∗*p* < 0.001. (C) On-target editing efficiency for all 19 amino acid substitutions at V1103 across three genomic targets by amplicon sequencing. Residues marked in red (Phe, Tyr, Trp, His) indicate side chains containing aromatic rings. Amino acids shown in blue have aliphatic side chains. Data represent mean ± SEM from n = 3 biological replicates. (D) Four classes of amino acid structural modeling performed at position 1103 using ChimeraX (v.1.4). Residues colored gray are the mimic amino acids. The purple dotted lines represent spatial clashes in the structure. (E) GUIDE-seq off-target profiles for WT, V1103K, and V1103R at three initial screening sites (HEK293 SITE x2, RNF2-T6, GRIN2B-T3). Mismatched positions relative to the on-target sequence are highlighted in color. (F) Extended GUIDE-seq validation of V1103K across six additional genomic targets (FANCF-T3, RUNX1-T3, EMX1-T2, FANCF-T4, DYRK1A-T2, GRIN2B-T9).
  f. Use ChimeraX v.1.4 to visualize PISA-identified interactions with “distance” and “polar contacts” commands to confirm contact patterns.
    **CRITICAL:** PISA analysis provides quantitative interaction metrics that objectively identify primary DNA-contacting residues versus engineering-amenable positions. A residue with minimal DNA contact but adequate surrounding space represents an ideal engineering target.
3. Cavity Assessment at Engineering Target Position.
  a. Use CASTp server (http://sts.bioe.uic.edu/castp/) or similar cavity analysis tools to calculate pocket volume at the identified engineering target position.
  b. Evaluate cavity dimensions for engineering feasibility:
    i. Target volume: >50 Å^3^ can accommodate K or R substitutions.
    ii. Smaller cavities (30**–**50 Å^3^): May accommodate smaller residues (N, Q).
    iii. Minimal cavity (<30 Å^3^): Engineering may be challenging; alternative positions should be considered.
      ***Note:*** For FrCas9, V1103 resides in a cavity of approximately 63 Å^3^ (surface area 133 Å^2^), sufficient for accommodating lysine or arginine side chains.
  c. Visualize the spatial constraints using surface representation in ChimeraX v.1.4:
    i. Display the target residue with surrounding amino acids (5 Å radius).
    ii. Show DNA backbone to assess directionality toward +1 phosphate.
    iii. Identify potential steric constraints from neighboring residues.
  d. Document the cavity geometry with annotated structure images for reference.
    ***Note:*** Cavity analysis determines which amino acid substitutions are structurally feasible. The existing cavity can accommodate a larger, charged side chain that creates new phosphate interactions without disrupting protein architecture.
    ***Note:*** The PLL architecture differs among Cas9 orthologs. For example, SpCas9's 1107-KES-1109 element shows uniform contact distribution where all three residues form direct hydrogen bonds with DNA, whereas FrCas9 exhibits a “strong-strong-weak” pattern with the third position (V1103) positioned in a cavity adjacent to the +1 phosphate. This architectural difference affects engineering potential.

### Structure-guided variant library construction

**Timing: 5–7 days**

This section describes the construction of amino acid variant libraries at the identified PLL engineering target position through inverse PCR and seamless assembly. For comprehensive characterization of structure-activity relationships, all 19 possible amino acid substitutions can be generated to establish design principles transferable to other Cas9 systems. FrCas9 V1103 is used as a worked example throughout.

4. Inverse PCR Amplification of Entire Plasmid with Mutation.
  a. Design back-to-back primer pairs for each variant: primers anneal on opposite strands flanking the target position, with the forward primer containing the desired codon mutation.
  b. Prepare 50 μL PCR reactions for each variant:

**Table:** PCR reaction master mix

Reagent	Amount
2× Phanta Max Master Mix	25μL
Forward Primer	2 μL (10 μM)
Reverse Primer	2 μL (10 μM)
pFrCas9 template	50ng
Nuclease-free water	To 50 μL

**Table:** PCR cycling conditions

Steps	Temperature	Time	Cycles
Initial Denaturation	95°C	3 min	1
Denaturation	95°C	15 sec	30
Annealing	55**–**60°C	15 sec	
Extension	72°C	30 sec/1kb	
Final extension	72°C	5 min	1
Hold	4°C	forever
  c. Verify PCR products on 1% agarose gel (expect ∼6 kb band for full-length plasmid) and purify using gel extraction or column purification kit to remove template and primers.
  d. Quantify purified linear PCR products using NanoDrop spectrophotometry:
    i. Measure absorbance at 260 nm for DNA concentration.
    ii. Check A260/A280 ratio (should be ∼1.8 for pure DNA).
    iii. Target concentration: 50**–**100 ng/μL for optimal seamless assembly efficiency.
      ***Note:*** Inverse PCR amplifies the entire plasmid while introducing the mutation, eliminating the need for separate vector preparation.
5. Seamless Circularization and Bacterial Transformation.
  a. Set up seamless assembly reaction: add 100 ng purified linear PCR product to equal volume (typically 10 μL total) of 2×MultiF Seamless Assembly Mix (ABclonal).
  b. Incubate at 50°C for 15 min to circularize the plasmid through homology-based recombination at primer overlap regions.
  c. Transform 5 μL assembly product into 50 μL competent DH5α cells by heat shock according to the manufacturer protocol (ice 30 min → 42°C 30 s → ice 2 min).
  d. Add 950 μL SOC medium and shake at 37°C, 200 rpm for 1 h for cell recovery.
  e. Plate 100**–**200 μL on LB agar containing appropriate antibiotic (ampicillin 100 μg/mL or kanamycin 50 μg/mL) and incubate 12**–**16h at 37°C.
  f. Pick 3**–**4 colonies per construct for verification.

***Note:*** The seamless assembly mix recognizes 15**–**25 bp overlaps at primer ends, circularizing the linear PCR product without leaving scars.

6. Sequence Verification of All Variants
  a. Perform colony PCR using flanking primers or miniprep isolation (QIAprep Spin Miniprep Kit) from selected clones.
  b. Submit samples for Sanger sequencing using primers spanning the target region (recommend sequencing window of ±7**–**10 residues around the mutation site).
    ***Note:*** For FrCas9, use primers spanning residues 1095**–**1110 to confirm the mutation region.
  c. Verify the presence of desired mutation at V1103, absence of additional changes, and seamless junction quality.
  d. Perform midiprep (QIAGEN Plasmid Midi Kit) on sequence-confirmed clones to obtain >1 μg/μL plasmid DNA.
  e. Store verified plasmids at −20°C with proper labeling indicating the Cas9 ortholog and specific mutation.
    ***Note:*** For FrCas9, label as pFrCas9-V1103K, pFrCas9-V1103R, etc.
    **CRITICAL:** Sequencing both forward and reverse directions across the mutation site ensures high-confidence verification and detect any PCR-introduced errors.

### Cell transfection for on-target assessment

**Timing: 3–4 days**

This section describes functional screening of Cas9 variants through plasmid transfection in HEK293T cells to evaluate on-target editing efficiency.

7. HEK293T Cell Preparation.
  a. Culture HEK293T cells in DMEM + 10% FBS + 1% penicillin-streptomycin at 37°C, 5% CO_2_.
  b. Maintain cells between passages 5**–**15 in logarithmic growth phase.
  c. Seed cells in 24-well plates at 1×10^5^ cells/well one day before transfection.
  d. Verify 70**–**80% confluency on the day of transfection.

**CRITICAL:** Cell passage number and confluency significantly affect transfection efficiency. Use cells within passages 5**–**15 and ensure 70**–**80% confluency for optimal results.

8. Plasmid Transfection.
  a. Prepare DNA mixture: 500 ng Cas9 variant plasmid + 500 ng sgRNA plasmid in 50 μL Opti-MEM per well.
  b. In separate tube, mix 2 μL Hieff Trans® Liposomal Transfection Reagent (Yeasen) with 50 μL Opti-MEM and vortex for 15 s.
  c. Incubate diluted transfection reagent at 25°C for 5 min.
  d. Add diluted transfection reagent to DNA mixture dropwise while gently mixing, then incubate at 25°C for 15**–**20 min to form DNA-lipid complexes.
  e. Add the transfection mixture dropwise to cells and gently rock plate for even distribution.
  f. Optional: Monitor cell morphology at 6**–**8 h post-transfection. Replace medium with 500 μL fresh complete medium if signs of cellular stress are observed (cell rounding, >10% detachment, vacuolization, or reduced confluence).

**CRITICAL:** Include wild-type Cas9, empty vector, and untransfected controls in every transfection experiment to enable proper normalization and quality assessment.

***Note:*** The low-toxicity transfection reagents used in this protocol typically do not require routine medium replacement. Medium replacement serves primarily as a troubleshooting option for cases where specific conditions induce cellular stress.

9. Incubation and Genomic DNA Extraction.
  a. Incubate transfected cells at 37°C, 5% CO_2_ for 72 h, monitoring cell health and confluency daily.
  b. After 72 h, aspirate medium and wash once with PBS.
  c. Extract genomic DNA using TIANcombi DNA Lyse&Det PCR Kit (Tiangen) following the manufacturer’s protocol for cultured cells.
  d. Quantify DNA using NanoDrop spectrophotometer (target A260/A280 ratio: 1.8–2.0) and adjust concentration to 50**–**100 ng/μL for PCR amplification.
  e. Store extracted genomic DNA at −20°C for short-term use or −80°C for long-term storage.

### Amplicon sequencing for editing efficiency

**Timing: 4–5 days**

This section describes quantification of on-target editing efficiency through two-step amplicon PCR and high-throughput sequencing in 3 key steps.

10. Two-Step PCR Amplification of Target Sites.
  a. For first-step PCR, design gene-specific primer pairs flanking the target site to generate 200**–**250 bp amplicons (not exceeding 300 bp). Each primer contains a gene-specific region (18**–**25 bp) and an outer Illumina-compatible adaptor sequence (∼20 bp).
  b. Prepare 25 μL first-step PCR reactions as follows:

**Table:** PCR reaction master mix

Reagent	Amount
2× Phanta Max Master Mix (Dye Plus)	12.5 μL (2×)
Forward Primer (with adaptor)	1 μL (10 μM)
Reverse Primer (with adaptor)	1 μL (10 μM)
Genomic DNA	100 ng
Nuclease-free water	To 25 μL

**Table:** PCR cycling conditions

Steps	Temperature	Time	Cycles
Initial Denaturation	95°C	3 min	1
Denaturation	98°C	20 sec	25
Annealing	60°C	15 sec	
Extension	72°C	20 sec	
Final extension	72°C	5 min	1
Hold	4°C	forever
  c. Verify amplification by running 3 μL on 2% agarose gel (expect ∼240**–**290 bp including adaptors).
  d. Purify first-step PCR products using column purification kit or AMPure XP beads (1:1 ratio) to remove primers and primer dimers.
  e. For the second-step PCR, prepare 25 μL reactions using purified first-step products with dual-index primers as follows:

**Table:** PCR reaction master mix

Reagent	Amount
2× Phanta Max Master Mix (Dye Plus)	12.5 μL (2×)
Index Primer 1 (i7)	0.5 μM
Index Primer 2 (i5)	0.5 μM
Purified First-step Product	2 μL
Nuclease-free water	To 25 μL

**Table:** PCR cycling conditions

Steps	Temperature	Time	Cycles
Initial Denaturation	95°C	3 min	1
Denaturation	95°C	20 sec	11
Annealing	60°C	15 sec	
Extension	72°C	20 sec	
Final extension	72°C	5 min	1
Hold	4°C	forever
  f. Verify final library on 2% agarose gel (expect ∼290**–**340 bp including adaptors and indexes).
    ***Note:*** Two-step PCR strategy minimizes PCR bias while enabling efficient sample multiplexing with unique dual indexes. When designing amplicon primers, target the region centered on the sgRNA cut site (±100 bp), avoid repetitive sequences, and ensure at least 50 bp on each side of the cut site for comprehensive indel detection.
11. Library Quantification and Pooling
  a. Purify second-step PCR products using AMPure XP beads (0.8× ratio) to remove excess primers and select proper fragment sizes.
  b. Quantify purified libraries using Qubit dsDNA HS Assay Kit (target concentration: 5**–**20 ng/μL).
  c. Verify library size distribution and quality on Agilent Bioanalyzer or TapeStation (expect single peak at ∼300**–**350 bp).
  d. Calculate molarity of each library based on concentration and fragment size using online calculator (https://nebiocalculator.neb.com/).
  e. Pool libraries at equimolar concentrations to achieve final concentration of 4 nM for next-generation sequencing.

**CRITICAL:** Accurate quantification and equimolar pooling ensure balanced read distribution across all samples, preventing over- or under-representation of individual variants.

12. High-Throughput Sequencing and Data Analysis.
  a. Sequence pooled amplicon libraries on MGI-2000 platform using paired-end 150 bp reads, target a minimum of 10,000 reads per sample (20,000**–**50,000 reads recommended).
    Quality control and read processing
  b. Perform quality control of raw sequencing data using fastp 0.20.0 with default parameters to remove low-quality reads (Q < 20) and trim adaptors.
  c. Merge paired-end reads into single reads using FLASH software with default settings (minimum overlap: 10 bp, maximum mismatch density: 0.25).
  d. Analyze merged reads using CRISPResso2 software: upload processed FASTQ files, specify reference amplicon sequence, sgRNA sequence, and expected cut site position (±3 bp window).
    ***Note:*** CRISPResso2 aligns reads to genomic reference sequence and identifies all insertions, deletions, and substitutions at single-nucleotide resolution.
  e. Calculate editing efficiency as: (number of reads with indels within ±10 bp of cut site/total sequencing reads) × 100%.
  f. Calculate on-target locus efficiency to describe editing precision as: (number of on-target locus editing reads/total mutational reads) × 100%.
  g. Export editing profiles, mutation spectra, and quantification results for each FrCas9 variant. Generate visualization plots including allele frequency distributions, mutation position heatmaps, and pie charts of editing outcomes.
  h. Compile results into summary tables showing relative editing efficiency (normalized to wild-type FrCas9) for all 19 V1103 variants (see Table S3 for complete analysis results), the efficiency of D1101 and S1102 variants is summarized as well (Figures 1B and 1C).
    ***Note:*** CRISPResso2 provides comprehensive analysis including classification of editing outcomes (insertions, deletions, substitutions, and combinations), quantification of on-target modifications, and identification of predominant alleles. For publication-quality figures, export high-resolution PDF plots directly from CRISPResso2 output.

### Oligodeoxynucleotide design and synthesis

**Timing: 2–3 days**

This section describes preparation of double-stranded ODN (dsODN) for GUIDE-seq analysis in 3 key steps.

13. Design ODNs with Silent Mutations for GUIDE-seq Tagging.
  a. Design 34-bp dsODN tag: “GTTTAATTGAGTTGTCATATGTTAATAACGGTAT” following GUIDE-seq protocol specifications.
  b. Ensure tag sequence has no genomic matches in human genome using BLAST search.
  c. For editing ODNs, design 90**–**120 nt single-stranded templates with mutation at center position.
  d. Include 3**–**5 silent mutations around the target site to prevent gRNA re-cutting.

***Note:*** Silent mutations should maintain amino acid sequence while disrupting PAM or seed sequence recognition.

14. Synthesis and Quality Control.
  a. Order oligonucleotides from IDT DNA with standard desalting purification grade.
  b. Resuspend lyophilized oligos in nuclease-free water to 100 μM stock concentration.
  c. For dsODN preparation, mix complementary strands at equimolar ratio (50 μM each).
  d. Anneal by heating to 95°C for 5 min, then cool slowly to 25°C over 45 min.

**CRITICAL:** Proper annealing is essential for efficient dsODN integration. Slow cooling ensures complete duplex formation.

15. Preparation of Working Concentrations.
  a. Dilute annealed dsODN tags to 10 μM working stock in nuclease-free water.
  b. Prepare single-use aliquots (10**–**20 μL per tube) to avoid freeze-thaw degradation.
  c. Store aliquots at −80°C for long-term storage or −20°C for use within 2 weeks.
  d. Thaw on ice immediately before use and keep on ice during experimental setup.

### Cell electroporation for GUIDE-seq analysis

**Timing: 3–4 days**

This section describes GUIDE-seq tag integration for off-target detection is performed through electroporation in 3 key steps.

16. HEK293T Cell Preparation for Electroporation.
  a. Expand HEK293T cells to obtain sufficient numbers (minimum 5×10^6^ cells per condition).
  b. Use cells at 70**–**80% confluency, passages 5**–**15 for optimal viability and editing.
  c. Trypsinize cells, count using hemocytometer, and wash twice with PBS.
  d. Resuspend cell pellet in SF Nucleofector solution (SF Cell Line 4D-Nucleofector X Kit, Lonza, Cat# V4XC-2024) at density of 2×10^7^ cells/mL.
17. Multi-component Electroporation.
  a. Prepare electroporation mix per reaction: 2×10^5^ cells (10 μL), 2 μg Cas9 variant plasmid, 2 μg sgRNA plasmid, 200 pmol dsODN tag in SF Nucleofector solution.
  b. Transfer mixture to Nucleocuvette strip, avoiding bubbles.
  c. Electroporate using 4D-Nucleofector X Unit with program CM-130 (or optimized program for HEK293T cells per manufacturer’s guidelines).
  d. Immediately transfer cells to 500 μL pre-warmed DMEM in 24-well plate.

**CRITICAL:** Pre-warm culture medium to 37°C to minimize cell stress post-electroporation. Transferring cells to cold medium significantly reduces viability.

18. Incubation and Genomic DNA Extraction.
  a. Culture electroporated cells at 37°C, 5% CO_2_ for 48 h (optimal for tag integration).
  b. Assess cell recovery at 24 h by microscopic observation: Healthy cultures show >70% cell attachment with spread morphology consistent with the parental cell line and minimal floating debris. Poor recovery may indicate suboptimal electroporation conditions.
  c. Harvest cells by trypsinization, wash with PBS, and extract gDNA using high-quality kit.
  d. Verify DNA integrity on 0.8% agarose gel.
    i. Load 200**–**500 ng gDNA per lane with appropriate DNA ladder.
    ii. Run gel at 100V for 30**–**40 min.
    iii. Assess DNA quality: intact DNA appears as a single, tight band of high-molecular-weight DNA (>10 kb) near the well with minimal to no smearing, whereas degraded DNA appears as diffuse smearing extending down the gel, indicating DNA fragmentation.
    iv. Proceed only with samples showing intact DNA profiles.
  e. Quantify using NanoDrop (A260/A280 ∼1.8 for pure DNA); store at −20°C.

***Note:*** For users requiring quantitative viability assessment, Trypan Blue exclusion or automated cell counting can be performed on a parallel sample at the time of seeding or harvest.

### GUIDE-seq off-target profiling

**Timing: 4–5 days**

This section describes genome-wide off-target detection using GUIDE-seq methodology to identify dsODN tag integration sites across the genome.

19. GUIDE-seq Library Preparation.
  a. Set up first PCR (25 cycles) using tag-specific primers with 1 μg genomic DNA template.
  b. Purify first PCR products using AMPure XP beads at 0.8× ratio to remove primers.
  c. Perform second PCR (25 cycles) using 50 ng purified first PCR product to add Illumina adapters and indices.
  d. Size-select final libraries (200**–**600 bp) using AMPure XP beads at 0.9× ratio.
20. High-throughput Sequencing.
  a. Quantify libraries using Qubit dsDNA HS Assay (target: 2**–**10 nM).
  b. Verify size distribution on Bioanalyzer or TapeStation (expect peak at 300**–**400 bp).
  c. Pool libraries at equimolar concentrations and dilute to 2 nM for sequencing.
  d. Sequence on Illumina NextSeq or NovaSeq using paired-end 150 bp reads, targeting 1**–**5 million read pairs per sample.
21. Off-target Site Identification and Analysis.
  a. Download sequencing data and run GUIDE-seq pipeline (https://github.com/aryeelab/guideseq).
  b. Align reads to reference genome (hg38) and identify dsODN tag integration sites.
  c. Call off-target sites using threshold of ≥3 unique reads per site.
  d. Annotate sites for genomic location, calculate mismatch patterns, and determine on:off-target ratios (Figure 1E).

***Note:*** Sites with <3 reads are considered background and excluded from analysis.

### Data analysis and variant characterization

**Timing: 2–3 days**

This section describes comprehensive data integration and variant ranking to identify optimal PLL engineering candidates based on editing efficiency and specificity profiles.

22. Statistical Analysis of Editing Efficiency Data.
  a. Compile on-target editing data from amplicon sequencing for all variants across the initial screening sites.
    ***Note:*** For FrCas9, all 19 V1103 variants were tested across 3 genomic target sites.
  b. Calculate mean editing efficiency and standard error (SEM) for each variant with n≥3 biological replicates.
  c. Normalize all values to wild-type Cas9 editing efficiency at each corresponding site: relative activity (%) = (variant efficiency/WT efficiency) × 100%.
  d. Perform one-way ANOVA comparing all variants to wild-type Cas9, followed by Dunnett’s post-hoc test for pairwise comparisons.
  e. Apply Benjamini-Hochberg correction for false discovery rate control (FDR < 0.05) to account for multiple testing.
23. Structure-Activity Relationship Mapping and Design Principle Establishment.
  a. Categorize amino acid substitutions by chemical properties:
    i. Charge: K, R, H (positive); D, E (negative); others (neutral).
    ii. Hydrophobicity: A, V, L, I, M, F, W (hydrophobic); S, T, N, Q, K, R (hydrophilic).
    iii. Size: G, A, S (small); others (medium); F, Y, W, R, K (large).
  b. Generate heatmaps displaying relative activity (% of wild-type) for each substitution using GraphPad Prism or R.
  c. Map activity data onto Cas9 crystal structure using ChimeraX v.1.4 to visualize spatial-functional correlations at the target position and surrounding PLL environment (Figure 1D).
  d. Identify patterns from the saturation mutagenesis data and categorize substitution outcomes by side chain properties.
    ***Note:*** For FrCas9, linear side chains (L, K, R, C, N, Q) improved activity to 110**–**150% of WT; aromatic residues (F, Y, W) and proline reduced activity to 20**–**40% of WT; negatively charged (D, E) showed 40**–**60% of WT; small hydrophobic (A, V) maintained ∼100% WT activity.
  e. Based on the patterns identified above, prioritize positively charged residues with linear side chains (K, R) for PLL engineering, selecting K or R at analogous PLL positions based on cavity size and local electrostatic environment.
    ***Note:*** For FrCas9, V1103K emerged as optimal (30.15% fidelity improvement), with V1103R as secondary candidate; both K and R satisfy the design criteria of positive charge and linear geometry.
    ***Note:*** The saturation mutagenesis approach serves as a discovery tool to identify design rules rather than a requirement for every engineering project. Once these structure-activity principles are established through comprehensive characterization in one system, they can be applied to engineer other Cas9 orthologs efficiently through structure-guided rational design.
24. Identification of Optimal Variants.
  After completing on-target screening and GUIDE-seq experiments, the following analysis integrates both datasets to identify the lead variant—first narrowing candidates by editing efficiency and structure-activity relationships, then incorporating off-target profiles.
  a. Rank all variants based on average editing efficiency across the 3 initial target sites.
  b. Apply rational selection criteria informed by structure-activity analysis:
    i. Select variants showing ≥110% relative activity compared to wild-type.
    ii. Prioritize positively charged, linear side chain substitutions (K, R) identified as optimal in step 2e.
    iii. Exclude variants with known detrimental features (aromatic, negatively charged, or proline).
  c. Based on structure-activity principles and initial screening results, identify top candidates demonstrating superior on-target performance with favorable structural properties warranting comprehensive GUIDE-seq analysis.
    ***Note:*** For FrCas9, V1103K and V1103R emerged as top candidates (110-150% of wild-type activity), warranting GUIDE-seq analysis.
  d. Compare off-target profiles between top candidate variants at the 3 initial screening sites using GUIDE-seq results.
  e. Identify the lead variant based on:
    i. On-target activity: Maintained or enhanced editing efficiency (≥100% of WT) across all tested sites.
    ii. Off-target profile: Fewer off-target sites or reduced off-target frequencies in head-to-head comparison with other candidates.
    iii. Structure-activity consistency: Performance aligns with predicted structural features (charge, side chain geometry, cavity accommodation).
    iv. Specificity considerations: Conformational flexibility of the substituted residue may influence specificity outcomes.
      ***Note:*** For FrCas9, V1103K was identified as the lead variant over V1103R, as it maintained ≥100% WT activity across all sites with fewer off-target sites, likely attributable to lysine's greater conformational flexibility compared to arginine's rigid guanidinium group.
  f. For lead candidates, perform extended validation across additional genomic target sites (recommended: 6-9 total sites) to confirm robustness.
    ***Note:*** The stepwise validation strategy—initial screening→ rational selection GUIDE-seq analysis → extended validation—balances thorough characterization with experimental economy.

### Validation of PLL engineering principles in additional Cas9 orthologs

**Timing: 1 week**

This section describes application of PLL engineering principles to other Cas9 systems. EvCas9 and GsCas9 are used as validation examples demonstrating that the design principles enable efficient rational engineering without exhaustive mutagenesis.

#### Phase 1: Sequence and structural analysis

**Timing: 2–3 days**

25. Sequence Alignment of PLL Regions.
  a. Retrieve target Cas9 protein sequence from UniProt or NCBI database (e.g., EvCas9: Eubacterium ventriosum Cas9; GsCas9: Gemella sanguinis Cas9).
  b. Align with FrCas9 using Clustal Omega (https://www.ebi.ac.uk/Tools/msa/clustalo/) or MUSCLE algorithm.
  c. Identify the PLL motif corresponding to FrCas9 D1101-S1102-V1103 through sequence and structural alignment.
  d. Locate the position analogous to FrCas9 V1103 (the engineering target) within the identified PLL motif, noting the native amino acid at this position.
26. Structural Assessment and Validation.
  a. Obtain or predict structure:
    i. Download crystal structure from PDB if available (preferred).
    ii. If unavailable, generate AlphaFold3^7^ prediction for Cas9-sgRNA-DNA ternary complex
  b. Validate structure quality (critical for AlphaFold3 predictions):
    i. Check predicted alignment error (PAE) plot: low error (<5 Å) between protein and DNA regions indicates reliable prediction.
    ii. Verify DNA backbone maintains proper B-form geometry (∼10.5 bp/turn).
    iii. Inspect protein-DNA interface for clear major and minor groove definition.
    iv. For AlpahFold3 predictions: Ensure DNA position near +1 phosphate is stable and not distorted. If the non-target strand shows high predicted error or irregular geometry, the prediction may be unreliable for PLL analysis.
  c. Load validated structure into ChimeraX v.1.4 and locate the PLL element adjacent to the +1 phosphate of the non-target DNA strand.
  d. Measure distances between PLL residues and DNA phosphate backbone:
    i. Strong interactions: <3 Å (direct hydrogen bonds).
    ii. Moderate interactions: 3**–**5 Å (may include water-mediated contacts).
    iii. Weak/no interaction: >5 Å.
  e. Upload structure to PISA^8^ server (https://www.ebi.ac.uk/pdbe/pisa/) and analyze protein-DNA interfaces to identify residues forming hydrogen bonds and salt bridges with the +1 phosphate.

**CRITICAL:** Always validate AlphaFold3 predictions carefully, as nucleic acid positioning can be unreliable. If the non-target strand shows high predicted error or irregular geometry, the prediction may not be suitable for PLL analysis.

27. Candidate Position Selection.
  a. Identify positions analogous to FrCas9 V1103 using structural alignment:
    i. For EvCas9: N1102 corresponds to FrCas9 V1103.
    ii. For GsCas9: T1106 corresponds to FrCas9 V1103.
    iii. Look for positions within the PLL loop that show minimal direct DNA contact.
  b. Verify candidate positions meet key criteria:
    i. Residue is within the structurally defined PLL motif (typically 3**–**5 amino acid loop element).
    ii. Position is within 5**–**8 Å of the +1 phosphate (close but not in direct strong contact).
    iii. Flanking residues (±1**–**2 positions) show stronger direct contacts with phosphate backbone (analogous to FrCas9 D1101/S1102 pattern, where these residues form multiple hydrogen bonds while the target position shows minimal contribution).
    iv. PISA analysis confirms flanking residues contribute more hydrogen bonds/salt bridges than the candidate position.
  c. Use CASTp^9^ server (http://sts.bioe.uic.edu/castp/) to estimate cavity volume at candidate positions (target: >50 Å^3^ for accommodating K or R side chains, as established from FrCas9 V1103 cavity analysis).
  d. Design mutation based on position-specific characteristics:
    i. For EvCas9 N1102: Asparagine (polar, medium-sized) → Arginine (long, positively charged) yields N1102R.
      ***Note:*** N→R substitution introduces positive charge and extends side chain for enhanced phosphate interaction. Arginine is selected over lysine due to the larger native residue (asparagine) suggesting sufficient cavity space.
    ii. For GsCas9 T1106: Threonine (small, polar) → Arginine (long, positively charged) yields T1106R.
      ***Note:*** T→R substitution introduces positive charge and significantly extends side chain. Arginine's length (5 heavy atoms in side chain) provides optimal reach to +1 phosphate from this position.
    iii. General principle applied from FrCas9: R (arginine) or K (lysine) provides both extended side chain and positive charge for enhanced phosphate interaction, as validated by FrCas9 V1103K/R performance.
    iv. Key advantage of rational design: Unlike FrCas9 where all 19 substitutions were tested, the design principles enable direct selection of optimal mutations (R or K) without testing aromatic residues (F, Y, W), negatively charged residues (D, E), or proline—all of which were empirically shown to be detrimental in FrCas9 saturation mutagenesis.
    v. This targeted approach reduces the experimental burden from 19 variants to 1**–**2 rationally designed candidates per Cas9 system.

#### Phase 2: Visual structure analysis and mutation design

**Timing: 1 day**

28. Visual Assessment of Mutation Feasibility.
  a. Load candidate positions into ChimeraX visualization:
    i. Display EvCas9 N1102 and GsCas9 T1106 with surrounding environment (5 Å sphere).
    ii. Show DNA backbone with surface representation to visualize spatial constraints.
    iii. Display neighboring residues in stick format.
  b. Manually evaluate space availability:
    i. Use ChimeraX’s mutagenesis wizard to model N1102R and T1106R substitutions.
    ii. Rotate view to examine mutant side chains from multiple angles.
    iii. Look for obvious steric clashes: overlapping van der Waals surfaces.
    iv. Evaluate whether the modeled side chain fits within the available space without steric clashes.
      ***Note:*** Clear space where the side chain fits without distorting the backbone indicates a viable candidate. Tight fit requiring minor rotamer adjustments may still work but should be tested experimentally. Obvious clash with neighbors or DNA suggests considering an alternative mutation.
  c. Assess electrostatic environment visually:
    i. Color residues by charge (blue: positive, red: negative, white: neutral).
    ii. Check for nearby charged residues within 5-8 Å.
    iii. Evaluate whether the local electrostatic environment is compatible with introducing a positively charged residue.
      ***Note:*** Introducing K/R is favorable when existing negative charges are nearby, as these may form stabilizing salt bridges with the DNA backbone. If the position is surrounded by multiple positive charges, electrostatic repulsion may reduce benefit. If the position is in a hydrophobic pocket, consider neutral polar residues (Q, N) as alternatives.
  d. Evaluate orientation toward +1 phosphate:
    i. Measure distance from modeled K/R side chain terminus to nearest phosphate oxygen.
    ii. Assess the distance from the modeled K/R side chain terminus to the nearest phosphate oxygen.
      ***Note:*** <6 Å with favorable geometry indicates a good candidate likely to form direct contact. 6–8 Å may enhance electrostatics and is worth experimental testing. >8 Å or unfavorable angle suggests lower priority, as significant binding improvement is unlikely.
  e. Compare modeled mutations to FrCas9 V1103K structure to verify similar spatial arrangement and phosphate-targeting orientation.
    ***Note:*** This approach relies on structural visualization and chemical intuition rather than quantitative energy calculations. Manual inspection by experienced researchers remains the most reliable method for rational PLL mutation design, as computational predictions often show limited correlation with experimental outcomes for protein-DNA interfaces.
29. Finalize Mutation Strategy
  a. Document design rationale for each mutation:
    i. EvCas9-N1102R: Clear cavity, favorable geometry, analogous to FrCas9 V1103, arginine selected based on FrCas9 principle that long positively charged residues enhance activity.
    ii. GsCas9-T1106R: Adequate space after T→R substitution verified by modeling, positive charge directed toward phosphate, structural alignment confirms analogous position to FrCas9 V1103.
  b. Prepare for experimental validation:
    i. If visual analysis shows acceptable structure consistent with FrCas9 success criteria, proceed to Phase 3.
    ii. If severe issues detected (e.g., steric clashes similar to FrCas9 aromatic substitutions), consider alternatives: K instead of R (shorter side chain), or neutral polar residues (Q, N) if cavity is restrictive.
  c. Expected experimental outcome prediction based on FrCas9 principles:
    i. Both N1102R and T1106R should show ≥100% activity relative to wild-type based on meeting all structural criteria.
    ii. Specificity should be maintained or improved, as observed with FrCas9 V1103K.

**CRITICAL:** The key advantage of rational design is reducing experimental burden from 19 variants to 1**–**2 rationally designed candidates per Cas9 system, representing ∼10-fold reduction in workload while maintaining high success probability when structural criteria are met.

#### Phase 3: Experimental validation

**Timing: 3–5 days**

30. Variant Construction and Verification.
  a. Generate EvCas9-N1102R and GsCas9-T1106R mutants using inverse PCR and seamless assembly (follow STEP 2 protocol).
  b. Sequence verify entire Cas9 coding region to ensure no unintended mutations were introduced.
  c. Perform midiprep to obtain high-quality plasmid DNA (>1 μg/μL) for functional assays.
31. On-target Activity Assessment.
  a. Transfect HEK293T cells with variant plasmids and sgRNAs targeting 3**–**4 well-characterized genomic sites with validated wild-type activity (follow STEP 3 protocol).
  b. Include corresponding wild-type controls (EvCas9-WT and GsCas9-WT) in parallel transfections.
  c. Extract genomic DNA after 72 h and perform two-step amplicon PCR followed by MGI sequencing (follow STEP 4 protocol).
  d. Analyze editing efficiency using CRISPResso2^10^ and calculate relative activity: (variant editing efficiency/wild-type editing efficiency) × 100%.
  e. Expected outcomes based on published validation:
    i. EvCas9-N1102R: Enhanced editing efficiency compared to EvCas9-WT across tested sites (typically 110-140% of WT, similar to FrCas9 V1103K performance range).
    ii. GsCas9-T1106R: Enhanced editing efficiency compared to GsCas9-WT across tested sites (typically 110**–**140% of WT).
    iii. Both variants maintain activity across diverse target sequences, demonstrating robustness of the rational design approach.
32. Specificity Validation (Optional but Recommended).
  a. Select 2**–**3 target sites showing robust on-target enhancement (>120% of wild-type) for off-target analysis.
  b. Perform GUIDE-seq^11^ to compare off-target profiles of N1102R/T1106R variants versus their wild-type counterparts (follow STEP 6-7 protocols).
  c. Calculate on:off-target ratios to assess whether specificity is maintained or improved:
    i. On:off ratio = (total on-target reads/total off-target reads).
    ii. Compare variant ratios to wild-type ratios.
    iii. Expected outcome: Ratios should be ≥100% of wild-type, potentially showing improvement as observed with FrCas9 V1103K (30.15% fidelity enhancement).
  d. Document any changes in off-target spectrum: new sites, eliminated sites, or altered frequencies.
  e. If off-target activity increases unexpectedly, consider that the specific Cas9 context may differ from FrCas9—this highlights the importance of comprehensive validation even for rationally designed variants.

***Note:*** Successful enhancement in both validated systems (EvCas9 and GsCas9) demonstrates that design principles established from comprehensive mutagenesis are transferable to structurally similar Cas9 orthologs. The key success factors are: (1) high structural similarity in the PLL region, (2) presence of an engineering-amenable cavity at the analogous position, (3) adherence to established design principles. Individual structural assessment remains essential for each new target system.

## Expected outcomes

### Saturation mutagenesis results and structure-activity relationships

**Figure 2:**
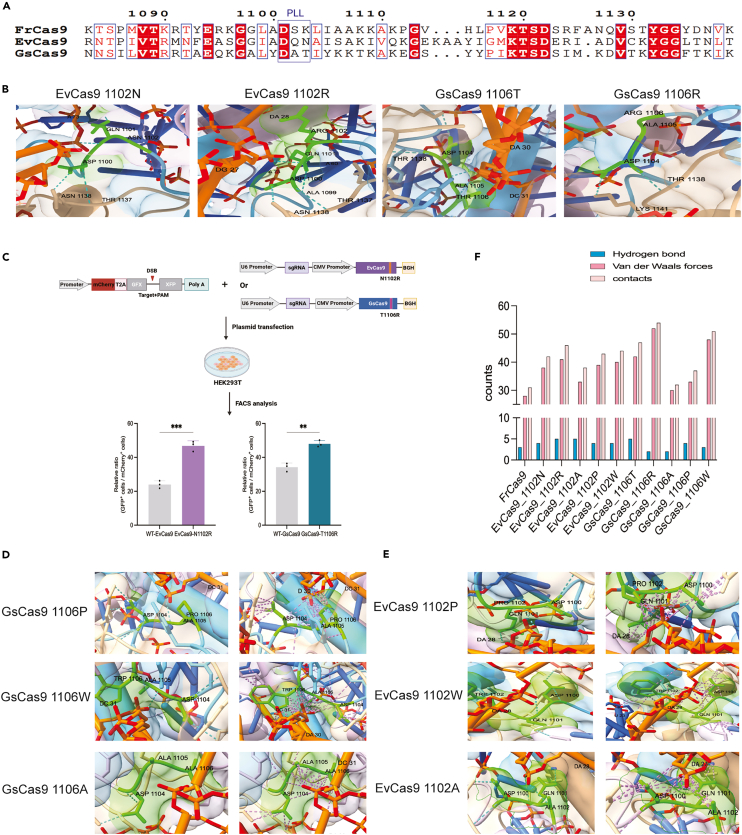
Cross-platform application of PLL engineering strategy (A) Sequence alignment identifying engineering targets (EvCas9 N1102, GsCas9 T1106) analogous to FrCas9 V1103. (B) Local spatial geometry of PLL domains showing wild-type (EvCas9 N1102, GsCas9 T1106) and arginine variants (EvCas9 N1102R, GsCas9 T1106R). Hydrogen bonds are indicated by blue dashed lines, and van der Waals forces are indicated by purple dashed lines. (C) Functional validation by SSA reporter assay improved editing efficiency for rationally designed variants compared to wild-type. Data represent mean ± SEM from n = 3 biological replicates. ∗∗*p* < 0.01, ∗∗∗*p* < 0.001. (D**–**E) Local spatial geometry comparison of mutations to other amino acid (Proline, Tryptophan, Alanine) at key sites (EvCas9 1102 and GsCas9 1106) in the PLL domain. Hydrogen bonds are indicated by blue dashed lines, and van der Waals forces are indicated by purple dashed lines. (F) Quantitative analysis of hydrogen bonding and van der Waals contacts for wild-type and variant residues across FrCas9, EvCas9, and GsCas9 systems.

Wild-type FrCas9 establishes the baseline for variant evaluation, with the PLL domain (D1101-S1102-V1103) positioned adjacent to the +1 phosphate of the target DNA strand (Figure 1A) and on-target editing efficiency varying by target site (Figures 1B and 1C). Saturation mutagenesis at V1103 reveals clear structure-activity patterns: linear side chain amino acids (K, R, L, C, N, Q) enhance editing efficiency to 110**–**150% of wild-type levels (Figure 1C), with V1103K achieving 30.15% overall fidelity improvement across nine target sites while maintaining or enhancing on-target efficiency (Figures 1E and 1F). In contrast, aromatic amino acids and proline reduce activity to 20**–**40% of wild-type due to steric clashes within the V1103 cavity, while negatively charged residues (D, E) show 40**–**60% activity due to electrostatic repulsion with DNA phosphate backbone (Figure 1C). These results validate three key principles: (1) cavity accessibility determines whether mutations fit within the structural constraints (Figure 1D); (2) positive charges (K, R) consistently outperform alternatives by strengthening DNA phosphate interactions; (3) linear extended side chains enable optimal backbone contact compared to branched or bulky structures.

### Cross-platform PLL engineering outcomes

When applying this protocol to new Cas9 systems, researchers can expect the following outcomes, as demonstrated with EvCas9 and GsCas9 (Figure 2). Sequence alignment and structural modeling identifies target positions analogous to FrCas9 V1103 (Figure 2A). For EvCas9 and GsCas9, N1102 and T1106 were identified as engineering targets showing minimal +1 phosphate contact (Figure 2B,). Computational modeling predicts that arginine substitutions (N1102R, T1106R) form enhanced hydrogen bonds with the DNA backbone (Figure 2B), providing structural rationale for mutation selection. Functional validation by Single strand annealing (SSA) reporter assay should demonstrate improved editing efficiency for rationally designed variants compared to wild-type (Figure 2C). Structure-activity analysis explains substitution outcomes: proline and aromatic residues cause steric clashes; alanine lacks electrostatic enhancement; only arginine optimally engages the phosphate (Figures 2D and 2E). These patterns mirror FrCas9 observations, validating the predictive design approach. Quantitative analysis confirms engineered variants form 2**–**3 additional hydrogen bonds and increased van der Waals contacts (Figure 2F). Successful enhancement is expected when target systems meet structural prerequisites: conserved PLL architecture, minimal target position DNA contact, strong flanking interactions, and adequate cavity space (>50 Å^3^). Individual structural validation remains essential for systems with divergent PLL geometries.

## Limitations

### Structural context dependencies

The effectiveness of PLL engineering varies significantly across the three different Cas9 orthologs tested in this work due to structural differences in the phosphate-binding cavity. While large Type II-A Cas9 systems (>1350 amino acids) typically respond favorably to lysine or arginine substitutions, smaller Cas9 variants or those with distinct PLL architectures may experience steric hindrance that reduces editing efficiency. Researchers working with Cas9 systems other than FrCas9 should perform structural analysis using available crystal structures or AlphaFold predictions to assess cavity geometry before implementing this protocol.

### Efficiency-specificity considerations

While the validated examples (FrCas9-V1103K, EvCas9-N1102R, GsCas9-T1106R) demonstrate both enhanced efficiency and maintained or improved specificity, individual results may vary depending on the target Cas9 system and genomic context. Some PLL modifications in other systems may present trade-offs between on-target efficiency and off-target activity. Researchers should comprehensively evaluate both on-target efficiency and GUIDE-seq off-target profiles to assess the overall performance of engineered variants for their specific applications.

## Troubleshooting

### Problem 1

Low or Variable Mutagenesis Efficiency (related to Step 4).

### Potential solutions

Poor mutagenesis success often results from inadequate primer design or low-quality template DNA. Redesign primers with 18**–**25 nucleotide flanking regions and balanced GC content. Use fresh, high-concentration template DNA (>100 ng/μL) and optimize annealing temperatures through gradient PCR (±5°C from calculated Tm). For difficult targets, consider overlap extension PCR as an alternative approach.

### Problem 2

Inconsistent ODN-Mediated Editing Results (related to Step 17).

### Potential solutions

Variable editing efficiency typically stems from inconsistent cell culture or electroporation conditions. Maintain HEK293T cells between passages 5**–**15 at 70**–**80% confluency. Include GFP controls to monitor electroporation efficiency. Store ODNs at −80°C in single-use aliquots and optimize electroporation parameters through pilot experiments testing different voltage/pulse combinations.

### Problem 3

Poor Amplicon Sequencing Quality or Low Read Counts (related to Step 10**–**12).

### Potential solutions

Sequencing issues often arise from PCR artifacts or insufficient template. Use high-fidelity polymerases with 25**–**30 cycles, gel-purify amplicons to remove primer dimers, and quantify libraries using fluorometric methods. Include no-template controls and increase sequencing depth to >10,000 reads per sample for low-activity variants.

### Problem 4

High Background Activity in Negative Controls (related to Steps 7**–**12).

### Potential solutions

Background editing indicates contamination or technical problems. Use dedicated pipettes and filter tips for each sample group. Include multiple negative controls (no-Cas9, no-gRNA, no-ODN) and process them first to minimize contamination. Sequence negative controls to identify contamination sources.

### Problem 5

Inconsistent Results Across Target Sites (related to Steps 10**–**15).

### Potential solutions

Target site variability may reflect chromatin accessibility or gRNA efficiency differences. Validate gRNA activity independently and include multiple target sites to account for site-specific variations. Consider chromatin accessibility data when interpreting results (suggested resources: ENCODE database at https://www.encodeproject.org/for ATAC-seq and DNase-seq data in common cell lines; or perform ATAC-seq on the specific cell type being used). Test alternative gRNAs for poorly performing targets.

## Resource availability

### Lead contact

Further information and requests for resources and reagents should be directed to and will be fulfilled by the lead contact, Bo Zhou (norman9614@126.com).

### Technical contact

Technical questions on executing this protocol should be directed to and will be answered by the technical contact, Zheng Hu (huzheng1998@163.com).

### Materials availability

This study did not generate any unique reagents. The commercially available reagents used in this study are provided in the key resources table.

### Data and code availability

This paper does not report original code. Any additional information required to reanalyze the data reported in this paper is available from the lead contact upon request.

## Acknowledgments

This work was supported by the 10.13039/501100001809National Natural Science Foundation of China, China (grant nos. 32171465 and 32371541).

## Author contributions

Conceptualization, Z.H. and B.Z.; methodology, M.Y., G.C., and J.X.; investigation, M.Y., G.C., J.X., and X.Z.; validation, J.X. and X.Z.; visualization, M.Y. and G.C.; writing – original draft, M.Y., G.C., J.X., and Z.H.; writing – review and editing, M.Y. and G.C.; supervision, Z.H. and B.Z.; funding acquisition, Z.H.; project administration, Z.H. M.Y., G.C., and J.X. contributed equally to this work. All authors have read and approved the final manuscript.

## Declaration of interests

The authors declare no competing interests.

## Supplemental information

Document S1. Table S1, S2, and S4

Table S3. Relative editing efficiency of all 19 V1103 variants across three target sites, related to step 23
