# Supplementary material for: Protocol for enhancing Cas9 efficiency and fidelity through structure-guided phosphate-locking loop engineering
Source: STAR Protoc. 2026 Mar 25;7(2):104452. doi: 10.1016/j.xpro.2026.104452 (PMC13052146; doi:10.1016/j.xpro.2026.104452)
Supplement: Document S1. Table S1, S2, and S4 [file mmc1.pdf]

Table S1. Primer sequences for FrCas9 V1103 saturation mutagenesis, related to Step 4.

Variant	Forward primer	Reverse primer
FrCas9-V1103P	GACAGCCCCCTGATCGCCGCCAAGAAG	GATCAGGGGGCTGTCTGCCAGTCCTCC
FrCas9-V1103F	GACAGCTTCCTGATCGCCGCCAAGAAG	GATCAGAAGGCTGTCTGCCAGTCCTCC
FrCas9-V1103Y	GACAGCTACCTGATCGCCGCCAAGAAG	GATCAGGTAGCTGTCTGCCAGTCCTCC
FrCas9-V1103W	GACAGCTGGCTGATCGCCGCCAAGAAG	GATCAGCCAGCTGTCTGCCAGTCCTCC
FrCas9-V1103H	GACAGCCACCTGATCGCCGCCAAGAAG	GATCAGGTGGCTGTCTGCCAGTCCTCC
FrCas9-V1103A	GACAGCGCCCTGATCGCCGCCAAGAAG	GATCAGGGCGCTGTCTGCCAGTCCTCC
FrCas9-V1103G	GACAGCGGCCTGATCGCCGCCAAGAAG	GATCAGGCCGCTGTCTGCCAGTCCTCC
FrCas9-V1103I	GACAGCATCCTGATCGCCGCCAAGAAG	GATCAGGATGCTGTCTGCCAGTCCTCC
FrCas9-V1103L	GACAGCCTGCTGATCGCCGCCAAGAAG	GATCAGCAGGCTGTCTGCCAGTCCTCC
FrCas9-V1103M	GACAGCATGCTGATCGCCGCCAAGAAG	GATCAGCATGCTGTCTGCCAGTCCTCC
FrCas9-V1103K	GACAGCAAGCTGATCGCCGCCAAGAAG	GATCAGCTTGCTGTCTGCCAGTCCTCC
FrCas9-V1103R	GACAGCAAGCTGATCGCCGCCAAGAAG	GATCAGCTTGCTGTCTGCCAGTCCTCC
FrCas9-V1103D	GACAGCGACCTGATCGCCGCCAAGAAG	GGAGGACTGGCAGACAGCGACCTGATC
FrCas9-V1103E	GACAGCGAGCTGATCGCCGC	GATCAGCTCGCTGTCTGCCA

	CAAGAAG	GTCCTCC
FrCas9-V1103C	GACAGCTGCCTGATCGCCGC CAAGAAG	GATCAGGCAGCTGTCTGCCA GTCCTCC
FrCas9-V1103T	GACAGCACCCTGATCGCCGC CAAGAAG	GATCAGGGTGCTGTCTGCCA GTCCTCC
FrCas9-V1103N	GACAGCAACCTGATCGCCGC CAAGAAG	GATCAGGTTGCTGTCTGCCA GTCCTCC
FrCas9-V1103Q	GACAGCCAGCTGATCGCCGC CAAGAAG	GATCAGCTGGCTGTCTGCCA GTCCTCC

Table S2. Primer sequences for amplicon sequencing library preparation, related to Step 10.

Site	sgRNA spacer	Forward Primer	Reverse Primer
GRIN2B-T3	TGGCCATGC GACCCTCTT CATA	ACACTCTTTCCCTACAC GACGCTCTTCCGATCTT CTGCTGCTCATTGGCTG AA	GTGACTGGAGTTCAGACG TGTGCTCTTCCGATCTAAG TGCTGGGATTACGGGTG
HEK293 SITE x2	GGTCATTGT CATGTCCAG TTGT	ACACTCTTTCCCTACAC GACGCTCTTCCGATCT AGCATCTTGACTCATC CTGCA	GTGACTGGAGTTCAGACG TGTGCTCTTCCGATCTACA TACATGCCCCTTTGCCT
RNF2-T6	ATGAGTTAC AACGAACA CCTCA	ACACTCTTTCCCTACAC GACGCTCTTCCGATCTT TTCCAGCAATGTCTCA G	GTGACTGGAGTTCAGACG TGTGCTCTTCCGATCTACA GAAGTCAGGAATGCTTGA ATA

Table S4. Sanger sequencing primers for FrCas9 variant verification, related to Step 6.

Primer Name	Sequence (5'→3')	Purpose
FrCas9-seq-F	ATCATCAGCCTGATCAATCAG	Sanger sequencing for V1103 verification
FrCas9-seq-R	CTGCGATCTTGGAGATCATG	Sanger sequencing for V1103 verification
